# Meta-analysis of association between caesarean section and postpartum depression risk

**DOI:** 10.3389/fpsyt.2024.1361604

**Published:** 2024-03-28

**Authors:** Jiajie Ning, Jing Deng, Shanshan Li, Caina Lu, Pingping Zeng

**Affiliations:** ^1^ Nursing College, Guangxi Medical University, Nanning, China; ^2^ Department of Neonatology, The First Affiliated Hospital of Guangxi Medical University, Nanning, China

**Keywords:** caesarean section, postpartum depression, emergency caesarean section, meta-analysis, systematic review

## Abstract

**Background:**

The association of caesarean section (CS) for postpartum depression (PPD) remains controversial. This study aims to explore the relationship between CS and the risk of PPD, in order to provide a basis for preventing PPD.

**Material and methods:**

We searched PubMed, Web of Science, Cochrane Library, and EMBASE databases for literature about the correlation between CS and PPD published as of February 2024. The combined odds ratios (ORs) and 95% confidence intervals (Cls) were obtained by flexible use of fixed-effects models or random-effects models.

**Results:**

A total of 18 publications were ultimately included in the analysis. Among these, 14 were cohort studies and 4 were case-control reports, encompassing 844,328 total cases. All of the included studies were deemed to be of moderate or higher quality. The meta-analysis indicated that the prevalence of PPD among parturients undergoing CS was 13.4% (95% CI: 6.5%-25.5%).The adjusted odds ratio (OR) for the association between CS and PPD was 1.12 (95% CI: 1.04-1.20) compared to the natural vaginal delivery (NVD) group. Specifically, the adjusted OR for the association between CS and PPD was 1.29 (95% CI: 1.18-1.40) during the first 1-6 months postpartum, and 1.23 (95% CI: 1.14-1.33) after 6 months postpartum. Furthermore, in comparison to the NVD group, the adjusted OR for elective caesarean section (ELCS) and emergency caesarean section (EMCS) were 0.96 (0.83, 1.10) and 1.20 (1.08, 1.34), respectively.

**Conclusion:**

Our findings suggest that PPD risk may rise in the presence of CS. In particular, the risk was increased by 20% in the EMCS group, and the risk of PPD within one to six months postpartum after CS increases by 6% compared to that at six months postpartum. In the future, more rational designs and in-depth studies are needed to obtain more accurate information.

**Systematic review registration:**

https://www.crd.york.ac.uk/PROSPERO/#recordDetails, identifier CRD42023389265.

## 1 Introduction

Postpartum depression (PPD) is one of the most common mental health issues affecting mothers after childbirth. Statistics indicate that the global incidence of PPD is as high as 10-20% (1). Typical symptoms include mood swings, sleep disturbances, loss of appetite, weight loss, apathy, and cognitive impairment. In severe cases, suicidal thoughts may also arise (2), posing a significant risk to the mother. Research has revealed that suicide accounts for nearly 20% of maternal deaths following childbirth (3). Furthermore, the negative impact of PPD can extend to the well-being of both the mother and the infant, affecting the mother’s physical and mental health, the mother-infant relationship, and marital bonds, while also increasing their risk of experiencing depressive episodes over the next 5 years (4, 5). Studies conducted in China and abroad have indicated that identifying risk factors early and implementing preventive interventions can effectively delay the onset and progression of PPD, thereby promoting the overall health and well-being of both the mother and the infant (6, 7).

Over the past few decades, the prevalence of caesarean section (CS) has significantly increased among women in both developed and developing countries, leading to heightened interest among public health researchers. These researchers have investigated the potential correlation between CS and PPD, although the nature of this correlation and whether it represents a direct link remain subjects of debate. Some researchers (8–11) have noted that the mode of delivery and the severity of PPD may not be related. Specifically, some researchers (8–10) have reported no discernible correlation between CS and PPD. A particular study (11) concluded that elective caesarean section (ELCS) showed no association with PPD, while emergent caesarean section (EMCS) was found to be significantly linked to PPD. This implies that unforeseen outcomes could impact the risk of PPD. An earlier report (12) suggested that women who underwent natural vaginal delivery (NVD) displayed more symptoms of PPD compared to those who had CS. At that time, the women in the study area harbored fear and anxiety regarding the painful experience of NVD. Nonetheless, a consensus has yet to be reached on this matter. Recent studies (11, 13–15) demonstrated a disparity in the incidence of PPD between CS and NVD, confirming that the occurrence of PPD was notably higher among women who underwent CS (especially EMCS), potentially due to biological or sociological factors. Furthermore, three meta-analysis studies (16–18) identified both types of CS as risk factors for PPD. However, one of these studies (17) did not find a statistically significant correlation between ElCS and the risk of PPD. This may be because the authors of the study only included data from 6 articles that included ELCS, and the study included information from 2016, which is somewhat outdated. Furthermore, one study (16) incorporated 25% of studies of inferior quality, whereas a different study (18) predominantly presented unadjusted results with a slightly reduced count of studies for effect sizes following consolidated adjustment, potentially resulting in a conclusion that lacks a certain degree of accuracy.

Therefore, this study conducted a meta-analysis on recent medium- to high-quality cohort and case-control studies. Women with different delivery types were grouped according to follow-up time in order to investigate the association between CS and PPD. The aim was to provide updated evidence-based information for early identification and effective prevention of maternal PPD for clinical health care providers. Additionally, subgroup analyses were performed to assess the correlation between other factors and CS or PPD.

## 2 Methods

This meta-analysis was reported according to the Preferred Reporting Items for Systematic Reviews and Meta-Analyses (PRISMA) (**Supplementary Table S1**) (19). This study has been registered on PROSPERO, and the registration number was CRD42023389265.

### 2.1 Search strategy

Literature related to the correlation between CS and PPD published prior to December 2022 was systematically gathered from databases such as PubMed, Web of Science, Cochrane Library, and EMBASE. To ensure that the latest research was not overlooked, an additional search and update was performed on February 27, 2024. The literature review involved a combination of subject terms and free-text keywords, with citations being followed to encompass a comprehensive scope. The subject terms utilized were “Caesarean section” and “postpartum depression”, and no restrictions were applied regarding language or publication date. Detailed strategies for the literature search can be found in **Supplementary Table S2**.

### 2.2 Inclusion and exclusion criteria

The inclusion and exclusion criteria for this study were strictly established based on the PICOS principles:

Participants: The subjects included in this study were parturients with PPD, regardless of race, region, whether they were primiparae and multiparae, or history of depression.

Intervention measures exposed by this study: parturient undergoing CS (including ELCS and EMCS). Studies in which PPD was managed using alternative therapies or treatments, such as diet, behavioral therapy, or antidepressants, were excluded.

Comparison group: parturients undergoing vaginal delivery (including NVD and assisted vaginal delivery (AVD).

Outcome: In the follow-up assessment, PPD scores of parturients were measured using self-report or clinician-administered measures. The number and probability of PPD in parturients with different modes of delivery were then calculated.

Type of study: cohort study and case-control study. Excluded were meta-analyses, clinical trials, reviews, editorials, preprints, animal studies, and other types of literature; Studies with ambiguous or incomplete data, which were not obtainable after contacting the authors, or studies in which data could not be transformed or combined, were excluded; Studies with a quality assessment score of ≤ 4 on the Newcastle-Ottawa Scale (NOS, **Supplementary Table S3**) (20) were also excluded.

### 2.3 Data extraction

The databases were independently searched by two researchers. They then proceeded to screen the titles and abstracts of the literature according to the predetermined inclusion and exclusion criteria. Subsequently, the full texts of the initially eligible literature were thoroughly examined. In cases of discrepancies, a third researcher was consulted for a final decision. Information extracted from the ultimately included literature encompassed the following: the first author’s name, year of publication, type and country of study, study duration, mode of delivery, proportion of CS deliveries, mother’s educational background, feeding method, sample size and age, tools used for depression measurement, outcome and duration of PPD, original odds ratio, adjusted odds ratio, and 95% confidence interval.

### 2.4 Quality evaluation of included literature

The quality of the included studies was assessed using the NOS, which comprised three dimensions and eight entries. These included research subject selection (4 entries, 4 scores), comparability between groups (1 entry, 2 scores), and outcome measurement or exposure factor measurement (3 entries, 3 points.) The total NOS score was 9. Studies with scores ≤4 were considered low-quality, those with scores of 5 to 6 were categorized as moderate-quality, and those with scores ≥7 were deemed high-quality. The quality evaluations were independently conducted by two researchers. In case of any disagreement, a third researcher intervened for discussion and resolution.

### 2.5 Publication bias

If the meta-analysis of the primary outcome (number of women with PPD by different modes of delivery) included 10 or more studies, funnel plots were adopted to seek possible publication bias and we visually observed whether there were any significant asymmetries. We planned to use the funnel plots to assess publication bias in the included literature.

### 2.6 Statistical analysis

Statistical analysis and meta-analysis were conducted using Stata v16.0, RevMan v5.3, and R v4.3.1. The correlation between CS and PPD was determined using OR (odds ratio) and a 95% confidence interval (CI) as the effect size, which were combined using the inverse variance method. The combined incidence and its corresponding 95% CI were utilized to analyze the occurrence of depression in the population. Heterogeneity was assessed using the I^2^ test. If I^2^ was less than 50%, the heterogeneity was deemed insignificant (16–18), and a fixed-effects model was employed. Conversely, a random-effects model was used. In cases of heterogeneity, sensitivity analysis and subgroup analysis were carried out to identify the sources of heterogeneity. Additionally, the publication bias of the included literature was examined using a funnel plot. A probability of less than 0.05 indicated statistical significance.

## 3 Results

### 3.1 Literature search results

**Figure 1:**
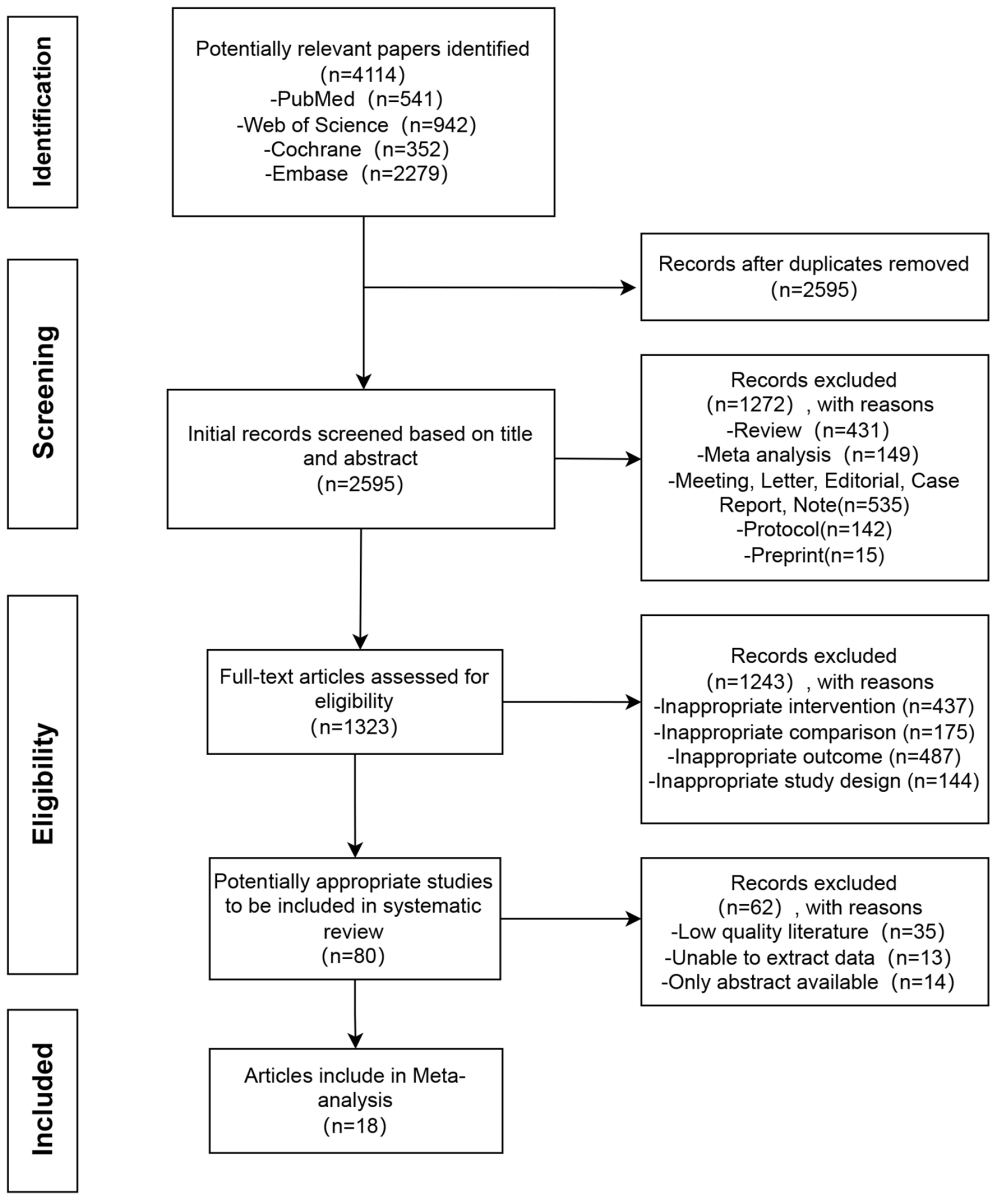
Flow chart of literature search.

The most recent search results have been selected and evaluated. Following the search process, a total of 541 articles from PubMed, 352 articles from Cochrane, 2,279 articles from EMBASE, and 942 articles from Web of Science were identified. Subsequently, 1,519 duplicate articles were excluded, leaving 2,595 unique articles for initial screening based on their titles and/or abstracts. Following this, the full texts of 1,323 studies were carefully reviewed and evaluated. After a further round of screening, 18 articles were deemed suitable for inclusion in the meta-analysis. A detailed depiction of the database search process is illustrated in **Figure 1**.

### 3.2 Basic features of included literature

A total of 18 studies (11, 21–37) were analyzed, involving 844,328 participants. The basic characteristics of the 18 included articles are listed in **Table 1**. Among these, seven studies were of moderate quality, while eleven were of high quality, as indicated in **Table 2**, which provides detailed evaluations of the literature’s quality. All 18 studies provided sufficient data to assess the correlation between CS and PPD. Among them, 8 studies (22–25, 33, 35–37) were adjusted for confounding factors. There were 14 cohort studies and 4 case-control studies, with the sample size ranging from 60 to 511,422 participants. Specifically, eleven studies (11, 24, 26–30, 32, 35–37) had <10,000 participants, while the remaining seven studies (21–23, 25, 31, 33, 34) had >10,000 participants. Furthermore, ten studies (21, 23, 26, 28–31, 34, 36, 37) were conducted in Asia, whereas seven studies (22, 24, 25, 27, 32, 33, 35) were carried out in Europe, one studies (11) were conducted in North America. Additionally, ten studies (11, 23, 24, 27–30, 34, 35, 37) utilized the Edinburgh Postnatal Depression Scale (EPDS) to measure PPD levels, two studies (22, 25) deployed the SCL-90, two studies (21, 31) adopted the 2010 LNHID, one study (26) used the CES-D, one study (36) employed the NHIS-NSC, one study (32) utilized the BDI, and one study (33) incorporated the HDR. The interventions varied in duration and length across the trials, and the assessment of PPD was conducted between 3 days and 12 months after delivery.

**Table 1:** Basic features of included studies.

Study	Country	Group	Number of subjects	Total number of subjects	age	history of depression(%)	Feeding type (%)	Mother’s education background	Follow up	Type of study	Adjusted Variable
T sai-Ching Liu, 2022 (21)	China	CS^a^/NVD^b^	10923/ 18869	32729	25-34 71.7%	10.9%	Not reported	Not reported	6-12 months	Cohort study	Not mentioned
Stina Kruse Skov, 2022 (22)	Denmark	CS^a^/NVD^b^/AVD^c^	4654/ 17914/ 4519	54474	20-39 98.2%	6.87%	Breastfeeding 92.44%	Not reported	6months	Cohort study	Based on maternal age, parity, year of delivery, BMI, smoking, alcohol consumption, physical activity, socio-occupational status and chronic diseases, pre-pregnancy mental health issues, and pregnancy mental health scores
Sachiko Baba, 2021 (23)	Japan	CS^a^/NVD^b^/AVD^c^	16802/ 51507/ 21615	89954	20-39 95.5%	5.08%	Bottle feeding/mixed feeding 57.76%	≥High School 94.08%	1-6 months	Cohort study	Age, parity, pre-pregnancy BMI, weight gain during pregnancy, gestational age at birth, newborn complications, marital status, education level, family income, antenatal mental status, history of mental illness, residential area, infant feeding method, etc
Patricia Eckerdal, 2019 (24)	Sweden	CS^a^/NVD^b^/AVD^c^	692/ 2872/ 324	3508	—	16.44%	Not reported	Not reported	6 weeks- 6 months	Cohort study	There were adjusted factors, but they were not specified
SS Adams, 2012 (25)	Norway	CS^a^/NVD^b^/AVD^c^	7181/ 43581/ 5052	55814	25-35 78.2%	Not reported	Not reported	≥Secondary 93.78%	6 months	Cohort study	Emotional distress at 30 weeks of pregnancy, parental education level, maternal age, etc
Shiow-Ru Chang, 2015 (26)	China	CS^a^/NVD^b^/	151/ 200	351	32.1 ± 0.3/ 33.9 ± 0.3	1.42%	Exclusively/mainly breastfeeding 81.66%	≥Secondary 99.14%	3 days- 12 months	Cohort study	Not mentioned
Rana Dousti, 2022 (27)	Iran	CS^a^/NVD^b^/	70/ 70	140	—	Not reported	Not reported	≥Diploma 77.14%	6 months	Cohort study	Not mentioned
Jamaan Alzahrani, 2022 (28)	Saudi Arabia	CS^a^/NVD^b^/	129/ 264	393	33.00 ± 7.72	9.92%	Breastfeeding 23.14% Bottle feeding 28.37% Both 48.48%	≥Secondary 91.56%	2 months	Cohort study	Not mentioned
Madhusmita Nayak 2020 (29)	India	CS^a^/NVD^b^/	100/ 100	200	18-35 96%	Not reported	Not reported	≥Secondary 62.00%	2 months	Cohort study	Not mentioned
Raneem SeifAl Nasr, 2020 (30)	Saudi Arabia	CS^a^/NVD^b^/	75/ 99	174	16-25 34% >25 66%	14.36%	Breastfeeding 77.93%	Not reported	2 months	Cohort study	Not mentioned
Jin Young Nam, 2017 (31)	Republic of Korea	CS^a^/NVD^b^/	29117/ 52330	81447	25-34 77.77%	0.47%	Continuous breastfeeding for 12 weeks 99.41% Discontinuous breastfeeding for 12 weeks 0.59%	Not reported	2 weeks	Cohort study	Not mentioned
MH Baghianimoghadam,2009 (32)	Iran	CS^a^/NVD^b^/	60/ 60	120	24.82 ± 5.20	Not reported	Not reported	Not reported	2 weeks	Cohort study	Not mentioned
Sari Raisanen, 2013 (33)	Finland	CS^a^/NVD^b^/AVD^c^	81711/ 420910/ 8801	511422	29 Mean	3.69%	Not reported	Not reported	6 weeks	Case-control study	Adjust for confounding factors that may affect the research results, such as maternal age, etc
Szu-Nian Yang, 2011 (34)	China	CS^a^/NVD^b^/AVD^c^	3676/ 6207/ 652	10535	29.73 Mean	Not reported	Not reported	Not reported	6 months	Case-control study	Not mentioned
Ann Josefsson, 2002 (35)	Sweden	CS^a^/NVD^b^/AVD^c^	50/ 267/ 79	396	29.6 Mean	Not reported	Breastfeeding 96.70%	Not reported	6 months	Case-control study	Family support, race, parental education level, child gender, planned delivery type, etc
Diana Petrosyan 2011 (36)	Armenia	CS^a^/NVD^b^/	66/ 269	335	≥25 63.3%	Not reported	Not reported	Not reported	1-3 months	Case-control study	Adjusted for current BMI, employment status, secondhand smoke exposure, child care anxiety scores, and self-esteem scores.
S. Smithson, 2020 (11)	America	CS^a^/NVD^b^	1680/ 370	2094	<35 80.6% ≥35 19.4%	Not reported	Not reported	Not reported	1-4 times	Cohort study	Not mentioned
Pratima Agarwal, 2023 (37)	India	CS^a^/NVD^b^	121/ 121	242	20-30 86.7% ≥31 13.3%	Not reported	Not reported	≥graduate 76.4%	1-6weeks	Cohort study	Sociological demographic characteristics

CS^a^, Caesarean section; NVD^b^, Natural vaginal delivery; AVD^c^, Assisted vaginal delivery.

**Table 2:** Literature quality evaluation.

Study	Entry 1	Entry 2	Entry 3	Entry 4	Entry 5	Entry 6	Entry 7	Entry 8	Total rating
T sai-Ching Liu 2022	Yes	Yes	Yes	Yes	Yes	No	Yes	No	7
Stina Kruse Skov 2022	Yes	Yes	Yes	Yes	Yes	No	Yes	No	7
Sachiko Baba 2021	Yes	Yes	Yes	Yes	Yes	No	Yes	No	7
Patricia Eckerdal 2019	Yes	Yes	Yes	Yes	Yes	No	Yes	No	7
SS Adams 2012	Yes	Yes	Yes	Yes	Yes	No	Yes	No	7
Shiow-Ru Chang 2015	Yes	Yes	Yes	Yes	Yes	No	Yes	Yes	8
Rana Dousti 2022	Yes	Yes	Yes	Yes	Yes	No	No	No	6
Jamaan Alzahrani 2022	Yes	Yes	Yes	Yes	Yes	No	No	No	6
Madhusmita Nayak 2020	Yes	Yes	Yes	Yes	Yes	No	No	No	6
Raneem Seif Al Nasr 2020	Yes	Yes	Yes	Yes	Yes	No	Yes	No	7
Jin Young Nam 2017	Yes	Yes	Yes	Yes	Yes	No	No	No	6
MH Baghianimoghadam 2019	Yes	Yes	Yes	Yes	Yes	No	No	No	6
Sari Raisanen 2013	Yes	No	No	Yes	Yes	Yes	Yes	No	6
Szu-Nian Yang 2011	Yes	No	Yes	Yes	Yes	Yes	Yes	No	7
Ann Josefsson 2002	Yes	Yes	Yes	Yes	Yes	Yes	Yes	No	8
Diana Petrosyan 2011	Yes	Yes	Yes	Yes	Yes	Yes	Yes	No	8
S.Smithson 2020	Yes	No	No	Yes	Yes	Yes	Yes	No	6
Pratima Agarwal 2023	Yes	Yes	Yes	Yes	Yes	Yes	Yes	No	8

Except for entry 5 which is 2 points, all other entries are 1 point. Details of the entries are given in **Supplementary Table S3**.

### 3.3 Meta-analysis results

#### 3.3.1 Incidence of PPD

We also constructed a forest plot to present the findings regarding the prevalence of PPD in the study population. The prevalence of PPD among the general parturient population was 11.7% (95% CI: 5.5%-23.3%, **Supplementary Figure 1**). The prevalence of PPD among parturients undergoing CS was 13.4% (95% CI: 6.5%-25.5%, **Supplementary Figure 2**), while the prevalence among those undergoing normal vaginal delivery was 10.4% (95% CI: 4.7%-21.2%, **Supplementary Figure 3**).

#### 3.3.2 Association between CS and PPD

**Figure 2:**
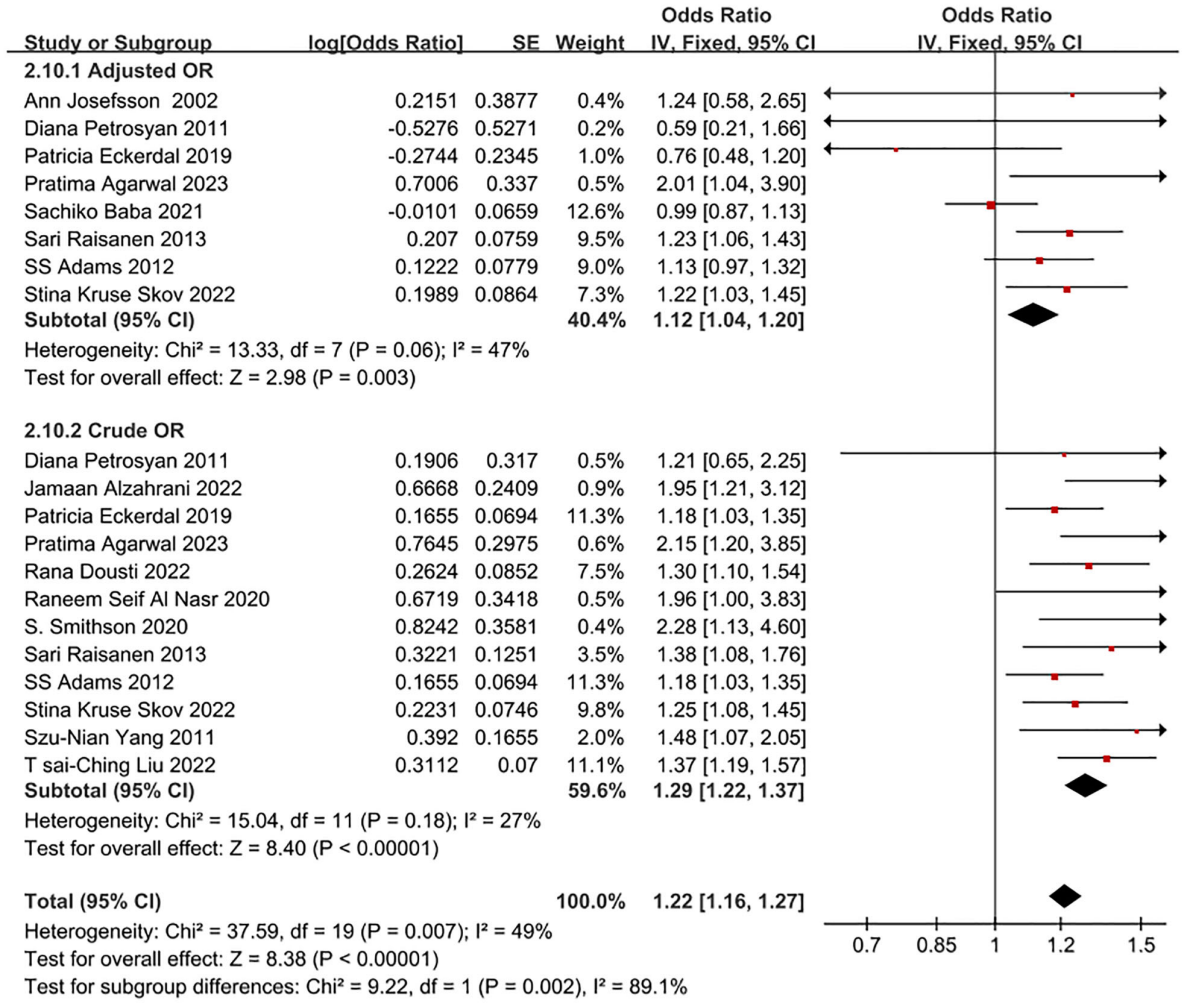
Forest plot of crude OR and adjusted OR for CS and NVD in PPD patients.

Eighteen eligible papers were included in the meta-analysis comparing CS and NVD. A fixed effects model was utilized to analyze the data. The findings indicated that the adjusted odds ratio for the association between CS and PPD was 1.12, with a 95% confidence interval of 1.04-1.20. See **Figure 2** for more details. All the studies included were of moderate to high quality, and there was no significant heterogeneity among them (I^2^ = 47%). Additionally, subgroup analysis was conducted to further compare based on clinical differences within the NVD group. This analysis considered factors such as the time dimension, different types of CS, race, region, primiparae and multiparae undergoing CS, and population size.

##### 3.3.2.1 Subgroup analysis by time dimension

**Figure 3:**
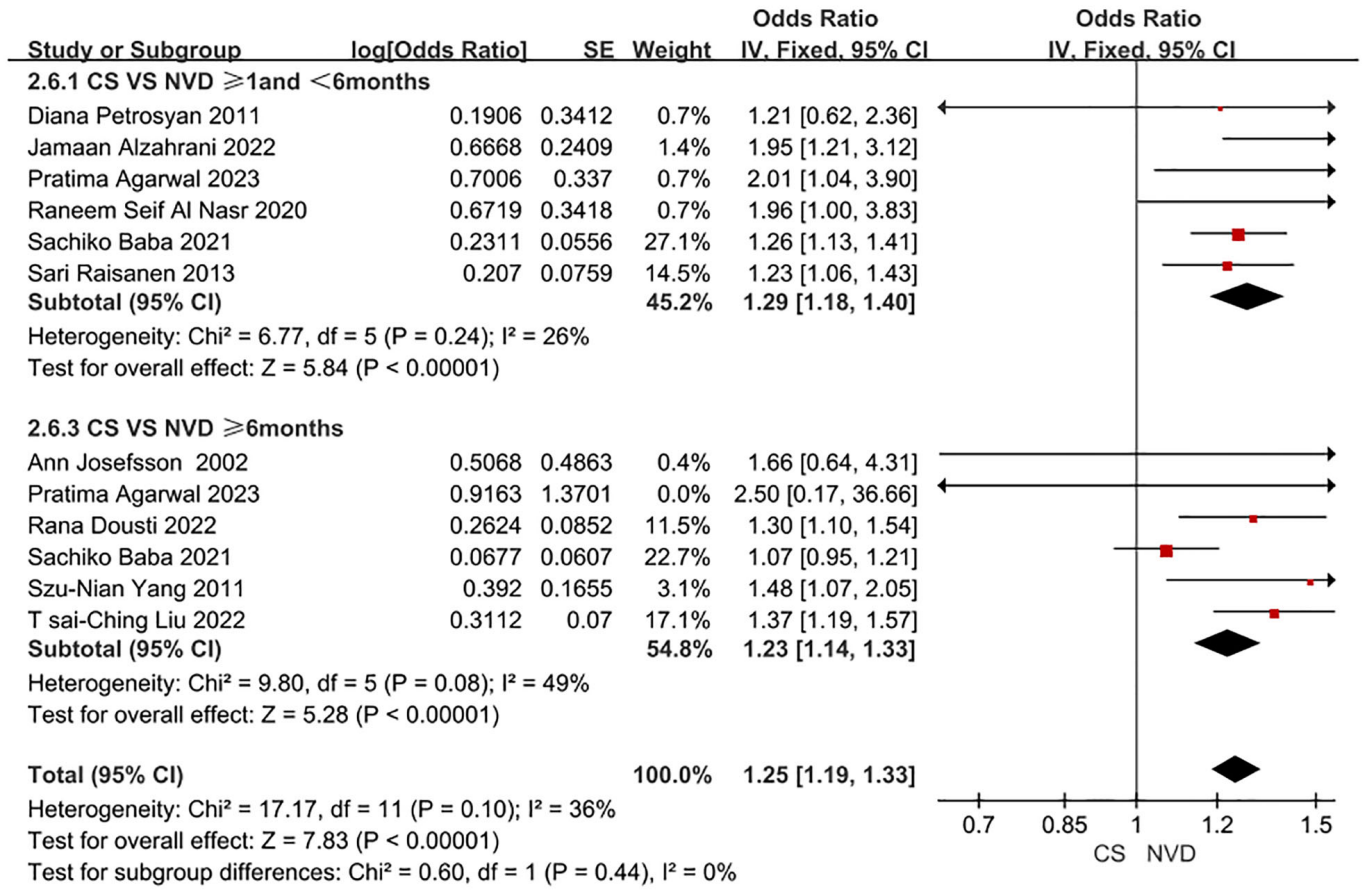
Forest plot of subgroup for PPD patient undergoing CS and NVD in time dimension.

In some studies (24), we discovered that the association between CS and the increased risk of PPD after one month was minimal compared to normal vaginal delivery (NVD). However, after adjusting for demographic factors, there was no discernible increase in PPD risk after six months postpartum. Therefore, we conducted a subgroup analysis, using postpartum six months as the time node. A total of 10 studies were included in this subgroup analysis by time dimension. The adjusted odds ratio (OR) for the association between CS and PPD within one to six months postpartum was 1.29 (95% CI: 1.18-1.40); after six months postpartum, the adjusted OR was 1.23 (95% CI: 1.14-1.33), as detailed in **Figure 3**. The data included in this subgroup analysis were all adjusted OR.

##### 3.3.2.2 Subgroup analysis by CS types

**Figure 4:**
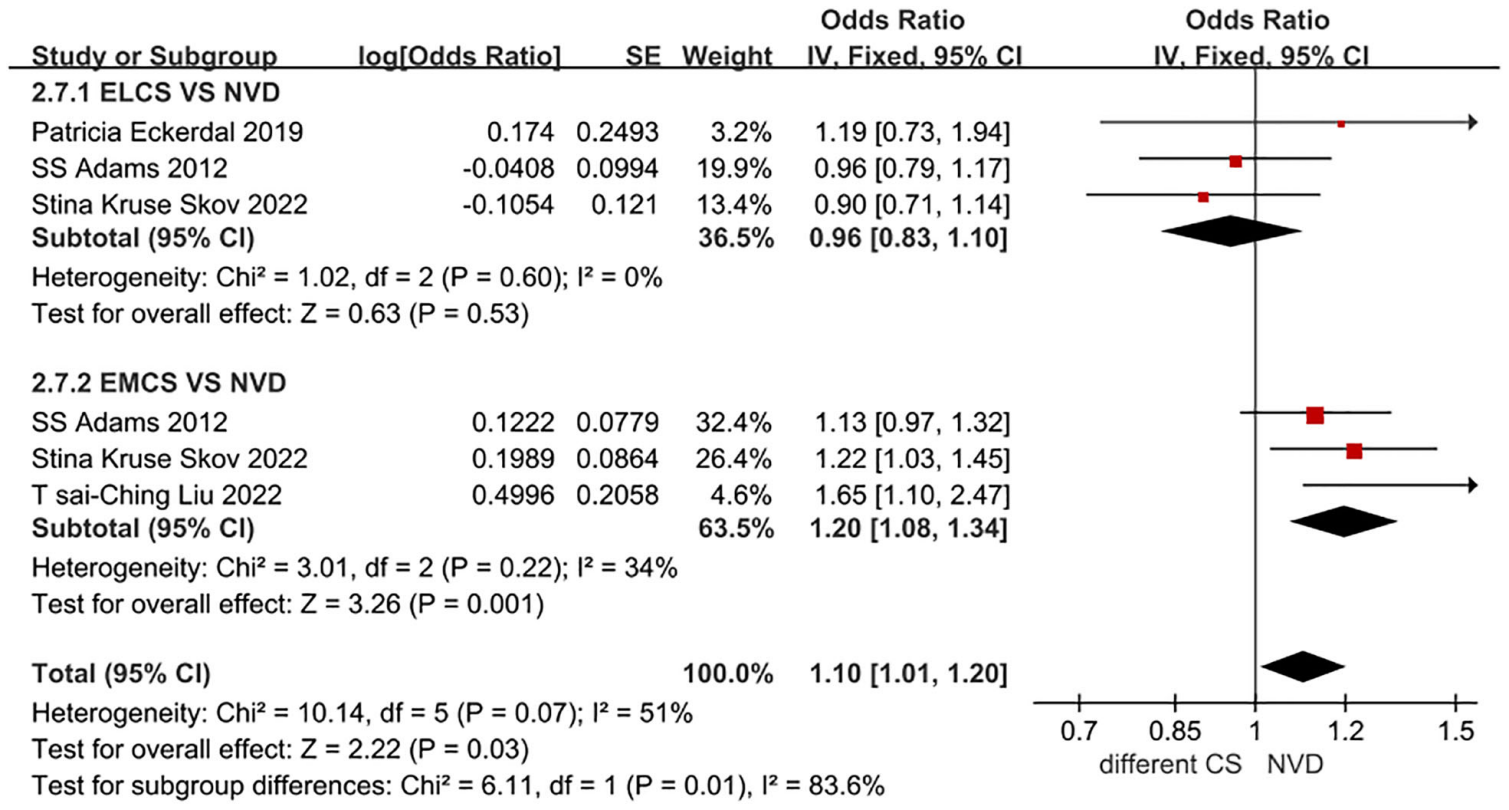
Forest plot for ELCS, EMCS and NVD in PPD patients.

In this subgroup analysis by CS type, a total of 4 studies were included. The adjusted OR for the association between ELCS and PPD was 0.96 (0.83, 1.10), and the adjusted OR for the association between EMCS and PPD was 1.20 (1.08, 1.34). as detailed in **Figure 4**. The data included in this subgroup analysis were all adjusted OR.

##### 3.3.2.3 Other subgroups

Although there was a high correlation between CS and PPD risk in all populations, we observed a stronger correlation in the Asian population (OR = 1.35, 95% CI: 1.17-1.57, I^2^ = 58%, P < 0.001). However, there was significant heterogeneity. In European populations (OR=1.17, 95% CI: 1.06-1.30, I^2^ = 16%, P = 0.002), the association was relatively weak, but the heterogeneity was not significant (**Table 3**). The data included in this subgroup analysis all consisted of adjusted OR.

**Table 3:** Additional subgroup analyses of PPD risk based on the features of included studies.

Other study subgroups	Studies (articles)	OR(95%Cl)	I^2^(%)	P values
Region				
Asia	12	1.35(1.17-1.57)	58	<0.001
Europe	5	1.17(1.06-1.30)	16	0.002
North America	1	–	–	–
Sample size				
<10000	11	1.44(1.26-1.64)	0	<0.001
>10000	7	1.19(1.11-1.27)	24	<0.001

**Figure 5:**
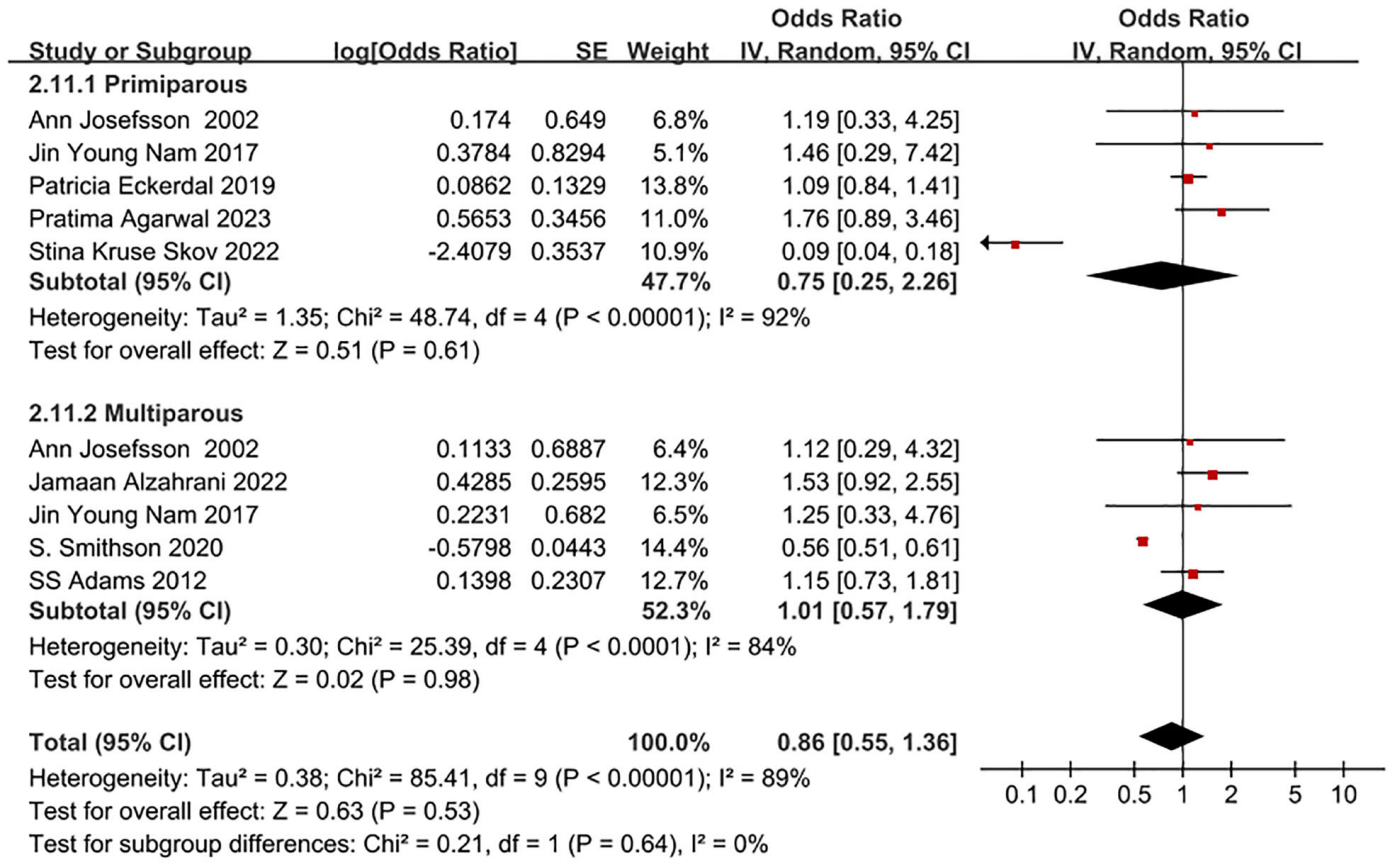
Forest plot of subgroup for whether PPD patient undergoing CS and NVD is primiparae.

Eight studies (11, 22, 24, 25, 28, 31, 35, 37) were included in the analysis, reporting the adjusted odds ratio (OR) for the incidence of PPD in primiparous women undergoing CS compared to multiparous women and those undergoing normal vaginal delivery (NVD). The OR for the occurrence of PPD in primiparous women undergoing CS was 0.75 (95% CI: 0.25-2.26, I^2^ = 92%, P = 0.61), while the OR for the occurrence of PPD in multiparous women undergoing CS was 1.01 (95% CI: 0.57-1.79, I^2^ = 84%, P = 0.98), as outlined in **Figure 5**. It’s important to note that the data included in this subgroup analysis were all adjusted OR.

The analysis of both CS and PPD in each of the seven largest studies (21–23, 25, 31, 33, 34) revealed that they accounted for more than 99% of the total cases in this meta-analysis, with a sample size exceeding 10,000. A weaker correlation (OR=1.19, 95% CI: 1.11-1.27, I^2^ = 24%, P<0.001) was observed between CS and PPD for the group with over 10,000 participants, compared to the group with fewer than 10,000 participants (OR=1.44, 95% CI: 1.26-1.64, I^2^ = 0%, P<0.001) (**Table 3**). Consequently, it can be inferred that the correlation between CS and PPD gradually diminished as the sample size increased.

### 3.4 Sensitivity analysis

The meta-analysis was performed again after excluding individual studies one by one, and the combined results revealed there were no significant changes, proving that the combined model was robust and reliable.

### 3.5 Publication bias

**Figure 6:**
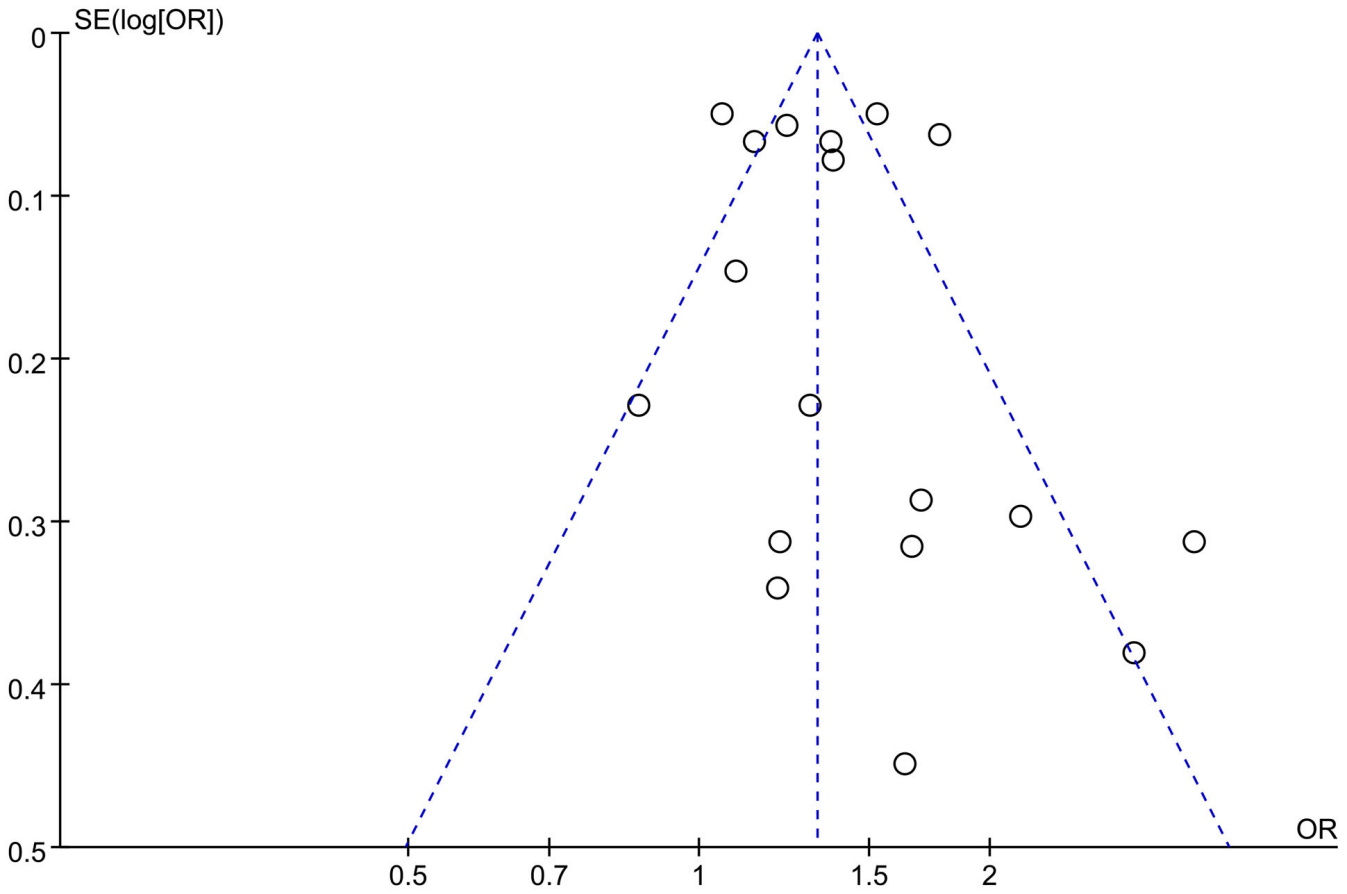
Funnel plot of publication bias for the correlation between CS and PPD.

Based on the number of mothers who eventually developed PPD in each group of the 18 included studies, a test for publication bias was performed, and the funnel plot showed that both sides were largely symmetrical, as shown in **Figure 6**. At the same time, based on the Egger bias test of this figure, p=0.820 was obtained. indicating no risk of publication bias.

## 4 Discussions

The research focused on examining the relationship between CS and PPD. This meta-analysis involved the gathering of data on the correlation between various delivery methods and different modes of CS in relation to PPD. It encompassed eighteen studies, involving 844,328 women from eleven different countries. A comprehensive search strategy utilizing subject terms in combination with free words across multiple databases was employed to encompass all available and eligible studies to date. Furthermore, the included studies comprised cohort and case-control studies of moderate quality and above, with the majority being published within the last five years. Additionally, the study categorized women with different delivery types based on PPD follow-up time and conducted various subgroup analyses to elucidate the association between CS and PPD. These approaches collectively strengthened the credibility of the study’s results.

Our study found that the incidence of PPD was 13.4% in parturients undergoing CS and 10.4% in those undergoing normal vaginal delivery (NVD). This indicates that CS represents a risk factor for PPD in parturients. It is well established that levels of steroid hormones, such as estradiol, progesterone, and cortisol, decrease significantly after delivery (38), irrespective of the mode of delivery. However, there has been a debate regarding the influence of steroid hormones on depression and PPD (39). Additionally, CS is linked to various maternal biological changes, including reduced prolactin levels and increased interleukin-6 levels, all of which are PPD risk factors (40). Furthermore, CS is associated with an increased risk of bleeding, which in turn elevates the likelihood of developing PPD (41). Societal perceptions and cultural beliefs associating “vaginal delivery” with “normal delivery” have also been reported to impact the psychological state of women undergoing CS, further contributing to the increased likelihood of PPD (42). These biological and psychological factors help explain why women who opt for CS are at a higher risk of experiencing PPD, thereby supporting the findings of our study. However, it should be noted that residual confounders were present in the pooled estimates due to some studies failing to adjust for confounding factors such as socioeconomic status and social support. While four high-quality studies reported that adjusted odds ratios reduced the risk of PPD in CS mothers by 17%, they had no impact on the overall outcome. This suggests that the adjustment of odds ratios did not affect the correlation between CS and PPD. Psychologically, many women may experience negative emotions such as low self-esteem, feelings of failure, loss of control, or disappointment following CS (22–25). The impact of wound care, slow postpartum recovery, and potential complications may further increase the risk of PPD. Additionally, women undergoing CS may lack confidence due to the inability to give birth naturally, even under necessary circumstances, which can create pressure in raising children. Furthermore, the physical pain and lifestyle changes after delivery can contribute to mental disorders, which may subsequently lead to PPD.

The study also examined the impact of CS mode, time dimension, primiparae and multiparae, and region on PPD risk. The subgroup analysis revealed that PPD risk increased by 20% in parturients undergoing EMCS, while it decreased in parturients undergoing ELCS. This difference may be attributed to the use of different types of anesthesia. Studies (43–47) have shown that women who undergo EMCS often receive general anesthesia, while those who undergo ELCS typically receive spinal anesthesia. General anesthesia has been associated with various biochemical mechanisms that could lead to the occurrence of PPD, such as inhibiting 5-HT uptake, reducing synaptic transmission of serotonin, dopamine, and norepinephrine, and interfering with the synthesis of S-adenosylmethionine. Additionally, women who deliver by EMCS may experience physical fatigue, along with the psychological burden and subjective feeling of being unable to give birth due to NVD failure. These factors can contribute to a negative delivery experience and potentially lead to depression. However, the results for the ELCS group were not statistically significant (P=0.55), possibly due to the limited number of study subjects (only 14,869 pregnant women who chose ELCS). Therefore, more rational designs and in-depth studies focusing on the effect of ElCS and EMCS on PPD risk should be conducted in the future. Our study does not report the original OR and adjusted OR data for the risk of PPD in women on AVD versus CS, which is one of our limitations. It is important to conduct more rational designs and in-depth studies in the future to confirm this conclusion.

Our subgroup analysis involved grouping by time dimension, using 6 months as the designated time node. This choice was made in order to provide a basis for further explanation. The findings of studies (24) indicated that the association between CS and the risk of PPD was minimal within the first month after delivery, as compared to normal vaginal delivery (NVD). However, upon adjusting for physical, socioeconomic, and psychological factors prior to delivery, the risk of PPD after 6 months postpartum was no longer significant. Additionally, studies (48, 49) have shown that women in Western countries, such as Ireland and Sweden, are entitled to at least 26 weeks (6 months) of maternity leave. Upon returning to work or taking care of their children, these women may face increased susceptibility to viral infections, which could contribute to elevated reports of anxiety and depression symptoms at 6 months. As a result, our studies utilized the time period of 6 months postpartum as the threshold for conducting subgroup analysis. Our study highlighted the potential for CS to elevate the occurrence of PPD within the first 1-6 months postpartum, indicating that the choice of CS may carry adverse emotional consequences for many women during the initial 6 months after delivery. Therefore, it is essential for healthcare professionals to recognize the need to impose certain limitations on the preference for CS, bearing in mind the subjective preferences of pregnant women and their clinical requirements.

Our study also revealed that compared to parturients undergoing vaginal delivery (NVD), the risk of PPD in parturients undergoing repeat CS was higher than that in parturients undergoing CS for the first time. A study (50) has shown that, in addition to considering other biopsychosocial factors, repeat CS is associated with greater pain severity, leading to an increased risk of PPD in parturients undergoing repeat CS, which corroborates the findings of the study we included (26). Studies (51, 52) have also indicated that the fear of pain and the delivery process may contribute to the occurrence of PPD in parturients undergoing repeat CS. During the early postpartum period, abdominal pain and incision scars from CS may result in dissatisfaction with their bodies, further increasing the risk of PPD in parturients undergoing repeat CS. Conversely, a study (53) has shown that the experience of the primary CS can alleviate the fear of pain in parturients, leading to lower pain scores for parturients undergoing repeat CS and thus reducing the occurrence of PPD. Early identification of the emotional changes of parturients in clinical practice and proactive management of their pain expectations may be beneficial.

In our subgroup analysis, we conducted a comparison between the European and Asian populations. It was found that the risk of PPD in Asian women undergoing CS is 18% higher than that in European women, when compared to NVD. This difference may be partly attributed to the practice of postpartum confinement, which is prevalent in many Asian communities, particularly in inland China and Taiwan, China. During postpartum confinement, mothers adhere to a high-protein diet and prolonged sitting, which can lead to negative body image issues. In contrast, countries like Europe and the United States do not observe the tradition of postpartum confinement (52). A previous study (54) has indicated that maintaining a positive body image can help prevent PPD. Similarly, Riquin et al. (55) have highlighted that dissatisfaction with body image during the perinatal period is linked to a higher risk of depression in mothers. Therefore, healthcare professionals should promote regular postpartum exercise to women, as this can help reduce postpartum weight retention and enhance body satisfaction, consequently lowering the incidence of PPD.

It is important to note that in this study, the Edinburgh Postnatal Depression Scale (EPDS) was used in 10 studies to assess the severity of PPD, while SCL-90 was used in 2 studies, LNHID from 2010 in 2 studies, CES-D in 1 study, NHIS-NSC in 1 study, BDI in 1 study, and HDR in 1 study. For more details, please refer to **Supplementary Figure 4**. The findings indicated that there were mostly no significant differences in the summarized results of groups that utilized different scales. We individually discussed each type of scale simultaneously. Notably, LNHID from 2010, NHIS-NSC, and HDR are all national health information databases, and the definition and diagnosis of PPD in parturients have been based on the content of “The Diagnostic and Statistical Manual of Mental Disorders” (DSM) from the American Psychiatric Association, which suggests that the screened data for PPD are similar. In a study (56) involving 1,831 pregnant women, the sROC curve for EPDS was found to be 0.90. The sROC curve for BDI was 0.91, indicating similarity to that of EPDS. Additionally, a separate study (57) revealed that the Cronbach’s α of CES-D was 0.84 and the Cronbach’s α of EPDS was 0.80, suggesting that the scales exhibited adequate reliability. Furthermore, the results of CFA in the same study indicated that the theoretical factorial structure of EPDS and CES-D can be sufficiently replicated. The three-factor structure of CES-D (“somatic symptom”, “negative impact”, and “anxiety”) and the three-factor structure of EPDS, including lack of enjoyment, anxiety, and depression, were also found to be replicable. In the case of SCL-90, a study (58) compared it with EPDS and indicated that their structures for evaluating depression were essentially similar. Research has shown that the results obtained from the various scales included in this study are similar, further indicating that the choice of PPD scale will not significantly impact the results. Thus, in this study, the merging of data from different scales is deemed reasonable and dependable. In future research, we will thoroughly consider the characteristics of the scales included in the study and endeavor to incorporate more data from the same scale for analysis.

### 4.1 Merits and limitations

This study provides comprehensive postpartum statistical data concerning pregnant women who have undergone various modes of delivery. The data includes the duration of PPD follow-up, demographic characteristics, and the tools used to measure PPD. A notable strength of this study is the large number of participants involved in the meta-analysis, totaling 844,328. This significantly enhances the statistical credibility and persuasiveness of the study, making it more likely to produce consistent results. These studies encompass extensive data on geographically diverse demographic factors and educational levels. The benefit of such a large participant pool is the ability to draw generalizable and consistent assessment results from a wide range of settings, populations, and behaviors (34). Furthermore, the studies included in this analysis consist primarily of cohort studies and case-control studies that are of moderate quality or higher. Most of these studies were published within the last five years. The majority of their findings have been adjusted to account for potential confounding variables, thereby strengthening the validity of the conclusions derived from the data.

The study has several limitations that need to be addressed. Firstly, by exclusively focusing on English literature, the study may introduce publication bias due to inadequate representation of literature. Secondly, following subgroup analysis, the study found small sample sizes and outcome indicators in each subgroup, necessitating further validation of the findings. Thirdly, the variation in evaluation criteria among different PPD assessment scales could potentially impact the analysis results. Additionally, maternal PPD may be influenced by factors such as duration, maternal age, and mode of CS, highlighting the need for further research. Lastly, the study’s inclusion of a limited number of individuals on AVD may affect the overall conclusions. Therefore, it is recommended that future prospective cohort studies or clinical trials with larger samples and higher quality be conducted to thoroughly examine the relationship between maternal CS and PPD and provide more robust evidence to support PPD prevention efforts. Given the limited quality of the statistical methods used in this study, its conclusions should be considered inconclusive.

## 5 Conclusion

The results of our study indicate a connection between CSs (especially EMCS) and the risk of developing PPD. It is evident that the risk of PPD in women undergoing CS is higher than in those undergoing Vaginal Delivery (NVD), and the risk of PPD within one to six months postpartum after CS increases by 6% compared to that at six months postpartum. Therefore, it is recommended that pregnant women make careful decisions regarding their mode of delivery and, when appropriate, seek the informed opinion of their healthcare provider. Moving forward, it is essential to carry out more systematic and comprehensive research in order to gain more precise insights.

## Data availability statement

The original contributions presented in the study are included in the article/**Supplementary Material**. Further inquiries can be directed to the corresponding author.

## Author contributions

JN: Conceptualization, Data curation, Formal analysis, Investigation, Methodology, Software, Validation, Writing – original draft. JD: Conceptualization, Data curation, Formal analysis, Investigation, Methodology, Software, Validation, Writing – original draft. SL: Conceptualization, Data curation, Formal analysis, Investigation, Validation, Writing – review & editing. CL: Data curation, Investigation, Methodology, Writing – review & editing. PZ: Conceptualization, Methodology, Supervision, Writing – review & editing.

## Supplementary material

Supplementary Figure 1 Forest plot of the incidence of PPD for parturient in the general population.

Supplementary Figure 2 Forest plot of incidence of PPD for parturient in the population undergoing CS.

Supplementary Figure 3 Forest plot of incidence of PPD for parturient in the population undergoing NVD.

Supplementary Figure 4 Forest plot of subgroup for PPD patient undergoing CS and NVD in different scales.

Supplementary Table 1 Preferred Reporting Items for Systematic Reviews and Meta-Analyses (PRISMA).

Supplementary Table 2 Database search strategy.

Supplementary Table 3 Newcastle-Ottawa Scale (NOS).
