# Supplementary figures and images for: Meta-analysis of association between caesarean section and postpartum depression risk
Source: Front Psychiatry. 2024 Mar 28;15:1361604. doi: 10.3389/fpsyt.2024.1361604 (PMC11006970; doi:10.3389/fpsyt.2024.1361604)

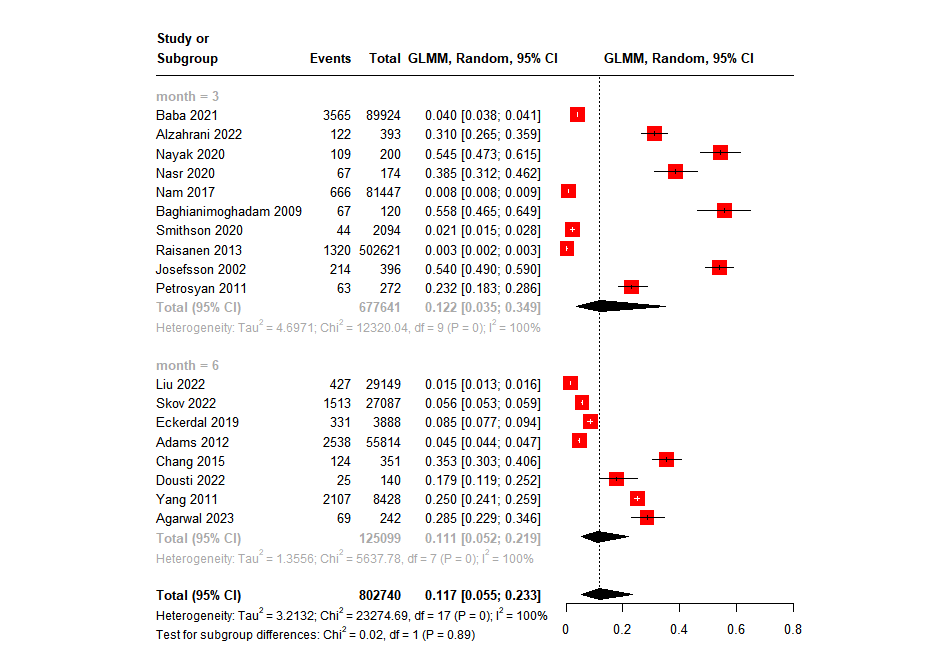

Supplement: Supplementary Figure 1 — Forest plot of the incidence of PPD for parturient in the general population. [file Image_1.tiff]

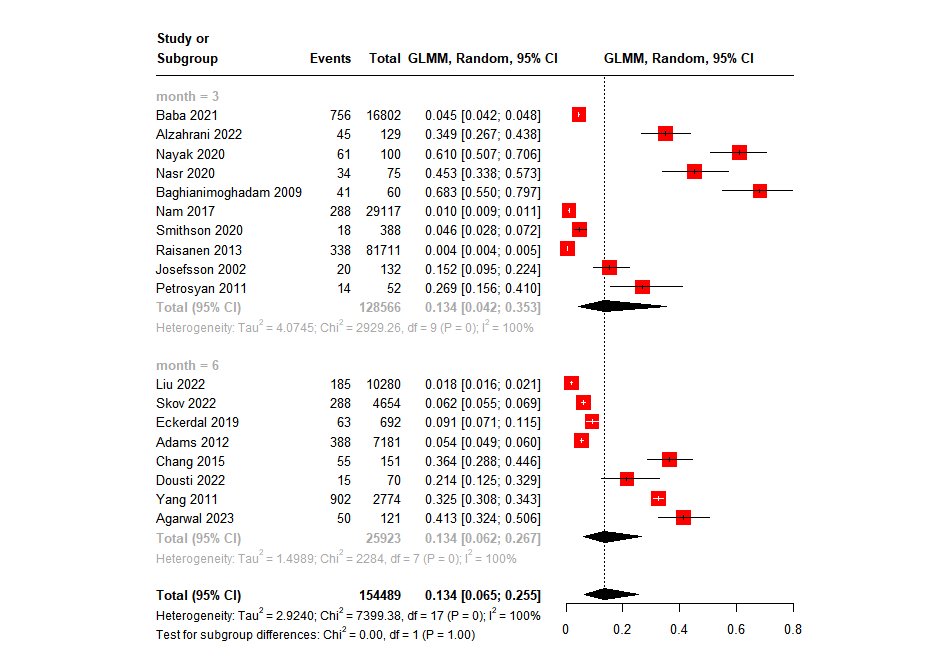

Supplement: Supplementary Figure 2 — Forest plot of incidence of PPD for parturient in the population undergoing CS. [file Image_2.tiff]

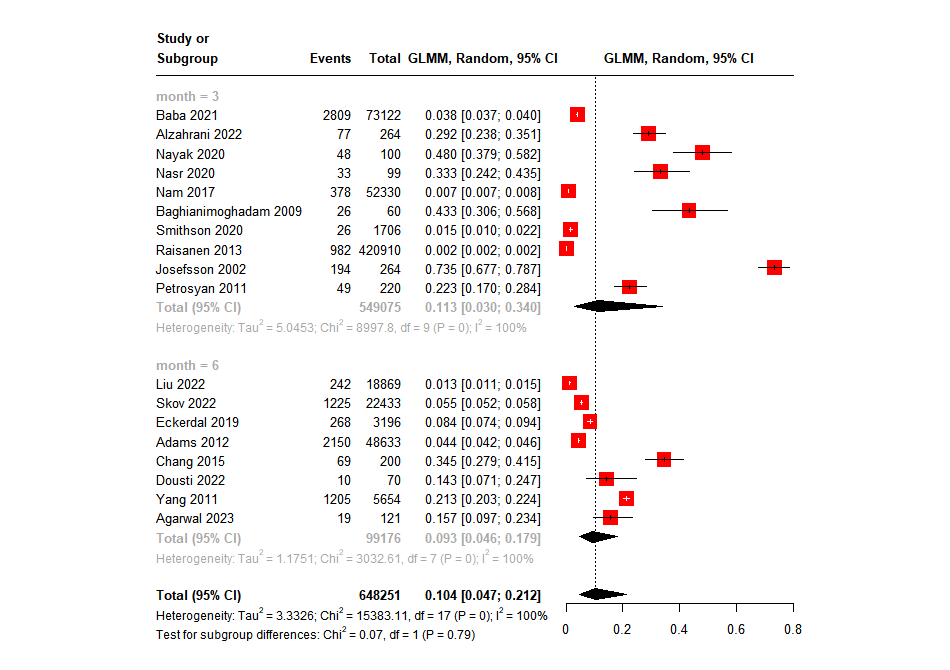

Supplement: Supplementary Figure 3 — Forest plot of incidence of PPD for parturient in the population undergoing NVD. [file Image_3.tiff]

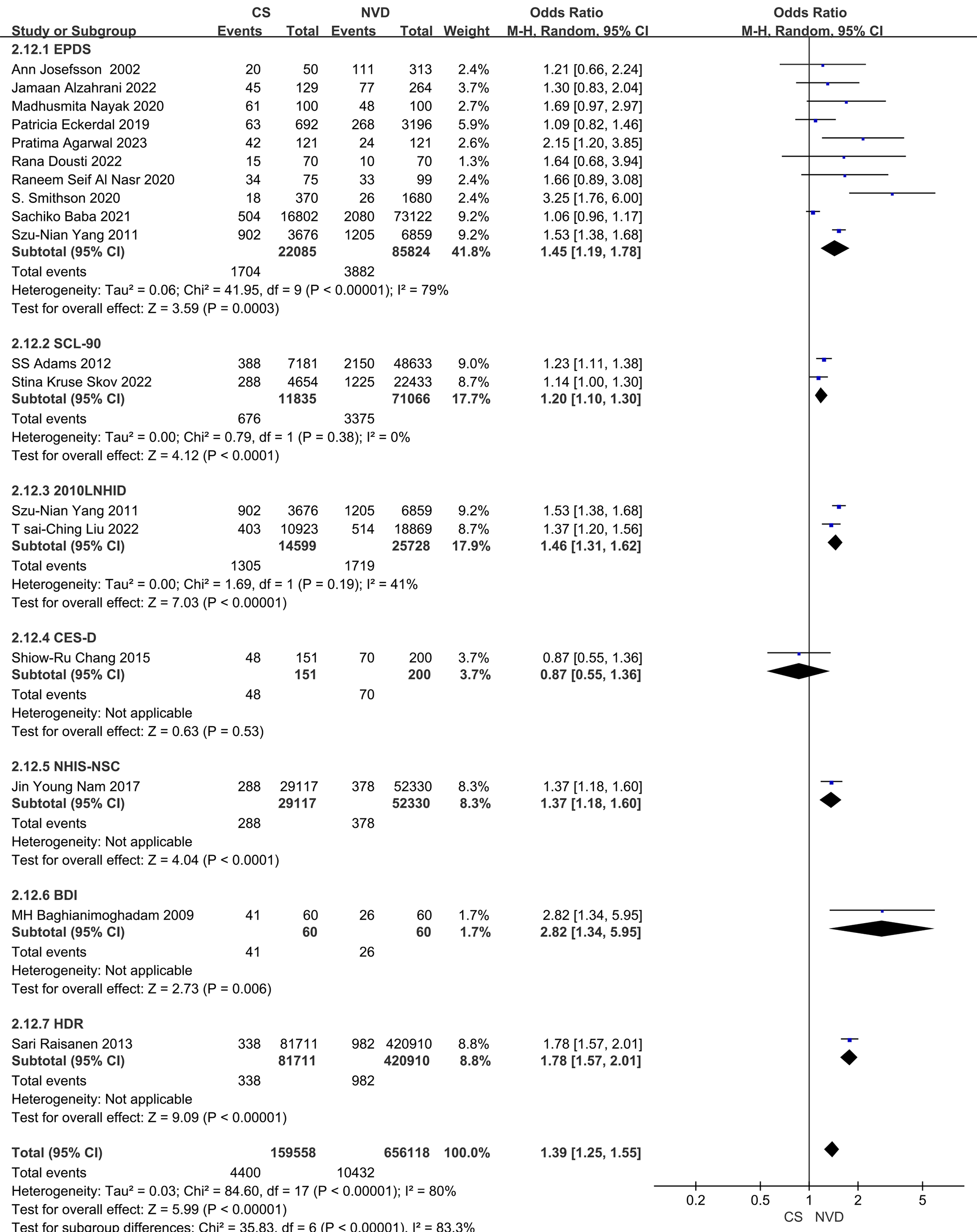

Supplement: Supplementary Figure 4 — Forest plot of subgroup for PPD patient undergoing CS and NVD in different scales. [file Image_4.tif]
