# Supplementary material for: Meta-analysis of association between caesarean section and postpartum depression risk
Source: Front Psychiatry. 2024 Mar 28;15:1361604. doi: 10.3389/fpsyt.2024.1361604 (PMC11006970; doi:10.3389/fpsyt.2024.1361604)
Supplement: Supplementary Table 2 — Database search strategy. [file DataSheet_2.pdf]

Table S2. Database search strategy.

1.pubmed Database search strategy

Search number	Query	Results
7	#3 and #6	541
6	#4 or #5	17,285
5	((((((((((((((((Postnatal Depression) OR (Depression, Postnatal)) OR (Post-Partum Depression)) OR (Depression, Post-Partum)) OR (Post Partum Depression)) OR (Postpartum Depression)) OR (Post-Natal Depression)) OR (Depression, Post-Natal)) OR (Post Natal Depression)) OR (Postnatal Dysphoria)) OR (Dysphoria, Postnatal)) OR (Post-Partum Dysphoria)) OR (Dysphoria, Post-Partum)) OR (Post Partum Dysphoria)) OR (Postpartum Dysphoria)) OR (Dysphoria, Postpartum)) OR (Post-Natal Dysphoria)) OR (Dysphoria, Post-Natal)) OR (Post Natal Dysphoria))	17,285
4	"Depression, Postpartum"[Mesh]	7,747
3	#1 or #2	84,651
2	((((((((((Cesarean Sections) OR (Delivery, Abdominal)) OR (Abdominal Deliveries)) OR (Deliveries, Abdominal)) OR (Caesarean Section)) OR (Caesarean Sections)) OR (Abdominal Delivery)) OR (C-Section (OB))) OR (C Section (OB))) OR (C-Sections (OB))) OR (Postcesarean Section))	84,651
1	"Cesarean Section"[Mesh]	54,164

2. Web of science Database search strategy

Search number	Query	Results
1	((((((((((TS=(Caesarean section) OR TS=(Cesarean Sections)) OR TS=(Delivery, Abdominal)) OR TS=(Abdominal Deliveries)) OR TS=(Deliveries, Abdominal)) OR TS=(Caesarean Section)) OR TS=(Caesarean Sections)) OR TS=(Abdominal Delivery)) OR TS=(C-Section (OB))) OR TS=(C Section (OB))) OR TS=(C-Sections (OB))) OR TS=(Postcesarean Section))	106511
2	((((((((((((((((((TS=(Postnatal depression) OR TS=(Postnatal Depression)) OR TS=(Depression, Postnatal)) OR TS=(Post-Partum Depression)) OR TS=(Depression, Post-Partum)) OR TS=(Post Partum Depression)) OR TS=(Postpartum Depression)) OR TS=(Post-Natal Depression)) OR TS=(Depression, Post-Natal)) OR TS=(Post Natal Depression)) OR TS=(Postnatal DysphTSia)) OR TS=(DysphTSia, Postnatal)) OR TS=(Post-Partum DysphTSia)) OR TS=(DysphTSia, Post-Partum)) OR TS=(Post Partum DysphTSia)) OR TS=(Postpartum DysphTSia)) OR TS=(DysphTSia, Postpartum)) OR TS=(Post-Natal	27868

	DysphTSia)) OR TS=(DysphTSia, Post-Natal)) OR TS=(Post Natal DysphTSia))	
3	#1 AND #2	942

- WOS: 1985 to 2024; - KJD: 1980 to 2024; - MEDLINE: 1950 to 2024; - SCIELO: 2002 to 2024 ;Date Run: Tue Feb 27 2024

3. Embase Database search strategy

No.	Query	Results
#3	#1 AND #2	2279
#2	'postnatal depression'/exp OR 'postnatal depression' OR (postnatal AND ('depression'/exp OR depression)) OR (depression, AND postpartum) OR (maternal AND depression) OR (depression, AND puerperium) OR (post AND partum AND depression) OR ('post natal' AND depression) OR (postpartum AND depression) OR (puerperal AND depression) OR (puerperium AND depression)	37565
#1	'caesarean section'/exp OR 'caesarean section' OR (caesarean AND ('section'/exp OR section)) OR (birth, AND abdominal AND operation) OR (caesarean AND birth) OR (birth, AND caesarean) OR (caesarian AND section) OR (caesarian AND birth) OR (cesarian AND section) OR (caesarean AND section) OR (cesarean AND delivery) OR (repeated AND cesarotomy) OR (sectio AND caesarea) OR fetectomy	148533

Date Run: 2024/2/27

4. Cochrane Database search strategy

ID	Search	Hits
#1	((((((((((Cesarean Sections) OR (Delivery, Abdominal)) OR (Abdominal Deliveries)) OR (Deliveries, Abdominal)) OR (Caesarean Section)) OR (Caesarean Sections)) OR (Abdominal Delivery)) OR (C-Section (OB))) OR (C Section (OB))) OR (C-Sections (OB))) OR (Postcesarean Section))	15911
#2	MeSH descriptor: [Cesarean Section] explode all trees	4474
#3	(((((((((((((((((Postnatal Depression) OR (Depression, Postnatal)) OR (Post-Partum Depression)) OR (Depression, Post-Partum)) OR (Post Partum Depression)) OR (Postpartum Depression)) OR (Post-Natal Depression)) OR (Depression, Post-Natal)) OR (Post Natal Depression)) OR (Postnatal Dysphoria)) OR (Dysphoria, Postnatal)) OR (Post-Partum Dysphoria)) OR (Dysphoria, Post-Partum)) OR (Post Partum Dysphoria)) OR (Postpartum Dysphoria)) OR (Dysphoria, Postpartum)) OR (Post-Natal Dysphoria)) OR (Dysphoria, Post-Natal)) OR (Post Natal Dysphoria))	3619
#4	MeSH descriptor: [Depression, Postpartum] explode all trees	976
#5	(#1 OR #2) AND (#3 OR #4)	352

Date Run: 27/2/2024
